# Editorial: Genotoxic pathways of reproductive outcomes

**DOI:** 10.3389/fcell.2025.1614251

**Published:** 2025-05-09

**Authors:** Irene Iavarone, Asok K. Dasmahapatra

**Affiliations:** ^1^ Department of Woman, Child and General and Specialized Surgery, University of Campania Luigi Vanvitelli, Naples, Italy; ^2^ Environmental Toxicology Division, Department of BioMolecular Sciences, School of Pharmacy, University of Mississippi, Oxford, MS, United States

**Keywords:** genotoxicity, reproduction, germ cell, oocyte, fertility

The concept of genotoxicity refers to the ability of certain compounds to cause damage to genetic material, leading to mutations, chromosomal aberrations, or cell death (Agarwa et al., 2015). Rearrangements might significantly impact reproductive health by affecting both male and female fertility. In humans, the exposure to genotoxic agents as heavy metals, pesticides, and environmental pollutants can lead to reduced sperm quality, increased rates of miscarriages, and developmental defects in offspring (Agarwa et al., 2015). In females, genotoxic agents can compromise oocyte quality and lead to chromosomal abnormalities or decreased reproductive potential (Krisher, 2004). Animal studies have also demonstrated the detrimental effects of genotoxicity on fertility, where exposure to chemicals like bisphenol A and other endocrine disruptors can affect reproduction, decrease birth rates, and cause developmental delays in offspring (Rhind et al., 2010). Genotoxicity may alter hormonal pathways, disrupt ovulation, and impair embryonic development, further affecting reproductive outcomes in both species.

The present Research Topic aims to provide an overview about the detrimental effects of the endocrine disruptors on multiple aspects of human and animal Reproductive health. In *“Elucidating the mechanisms and mitigation strategies for six-phthalate-induced toxicity in male germ cells”*, Kim et al. evaluate the cumulative effects of a mixture of Phthalate Esters on differentiated male germ cells. Indeed, they stimulate Reactive Oxygen Species generation and induce apoptosis. Moreover, Phthalate Esters are capable in inducing autophagy. Those effects have been demonstrated on a large sample clustering, and yet reverted using a triple inhibitor combination treatment.

In *“Obesity May Impair Response to Ovarian Stimulation. A Retrospective Observational Study on Oocyte Quality”*, Iavarone et al. focus on women’s Fertility outcomes basing on their Body Mass Index. Abdominal body fat distribution is important because anovulatory women have a greater waist circumference than ovulatory ones with equal Body Mass Index. In particular, they demonstrate that Anti-Müllerian Hormone levels are significantly decreased in obese women compared to Normal Weight and Overweight, thus emphasizing the importance of assessing weight in infertility’s treatment planning.

Zhang et al., in *“Corrigendum: Zearalenone Exposure Enhanced the Expression of Tumorigenesis Genes in Donkey Granulosa Cells* Via *The PTEN/PI3K/AKT Signaling Pathway”*, re-examine the toxicity of Zearalenone, a natural contaminant present in food and feed products which shows a negative impact on the reproductive potential of domestic animals in China. Their findings highlighted the deleterious influence of Zearalenone in inducing of Ovarian Cancer-related genes through the PTEN/PI3K/AKT signaling pathway in donkey’s granulosa cells *in vitro*.

In *“A Systematic Review of the Effects of Nanoplastics on Fish”*, Dasmahapatra et al. focus on plastics as a world-wide concern has been amplified due to their contamination in the environment, their capability to pass biological barriers in multiple organisms, thus polluting the environment. The most studied specie–e.g., Zebrafish–suffers from oxidative stress, decreased locomotor activities, altered immunity, lipid metabolism and neurotoxicity. Those are due to the progressive accumulation of nano-plastics during different stages of growth. In addition, they revised how nano-plastics imbalance steroidogenesis’ pathways.

Finally, in *“Differentially Expressed and Alternately Spliced Genes as a Novel Tool for Genotoxicity: A Computerized Study In ATT-Myc Transgenic Mice for the Recognition of Genotoxic and Non-Genotoxic Chemical”*, Alghamdi et al. study the application of hazardous chemicals on transgenic mice, showing that the most expressed gene after hybridization is identified as a new tumor suppressor gene in the context of hepatocellular carcinoma.

Taken together, with the growing exposure to environmental contaminants, studies on genotoxins are crucial in environmental toxicology as they can contribute to the development of diseases and other health-related problems in humans. Although the classification of genotoxins is a monumental tusk, to understand the mechanism of genotoxicity, the effects induced by pesticides, heavy metals, pharmaceutical products, persistent organic pollutants can be taken in to account. One of the basic mechanisms in toxicology is the activation of reactive oxygen or nitrogen species, which is involved from DNA damage to cellular functions. The articles published through the current Research Topic, “Genotoxic Pathways of Reproductive Outcomes” threw lights on mechanisms from autophagy to oxidative stress, techniques from *in vivo* to *in vitro*, models from cell lines to whole animals and chemicals from phthalates to Nanoplastics; therefore, the articles have significant impact to the readers, Researchers, and investigators involved in studying genotoxins.
